# Supplementary material for: Empirical methods for controlling false positives and estimating confidence in ChIP-Seq peaks
Source: BMC Bioinformatics. 2008 Dec 5;9:523. doi: 10.1186/1471-2105-9-523 (PMC2628906; doi:10.1186/1471-2105-9-523)
Supplement: Additional File 1 — A variety of html documents from the USeq web site detailing the available applications, their best usage, output file type descriptions, command line menus, etc. [file 1471-2105-9-523-S1.zip › USeqUserGuides/JavaDoc.zip!/JavaDoc/util/bio/seq/package-frame.html]

util.bio.seq


util.bio.seq

Classes 
  
BinaryBooleanReaderWriter
  
ConvertFasta2GCBoolean
  
ConvertFasta2RealSeqBoolean
  
FetchIndexedSequence
  
IndexedSequence
  
IndexFastas
  
IndexRegions
  
LowerCase2Xs
  
MakeSpliceJunctionFasta
  
MakeTranscriptome
  
MaskExonsInFastaFiles
  
MergeFastas
  
OligoTiler
  
ParseSeqsFromUCSCGeneTable
  
Seq
